# In Cellulo Mössbauer and EPR Studies Bring New Evidence to the Long‐Standing Debate on Iron–Sulfur Cluster Binding in Human Anamorsin

**DOI:** 10.1002/anie.202102910

**Published:** 2021-05-24

**Authors:** Sara Matteucci, Francesca Camponeschi, Martin Clémancey, Simone Ciofi‐Baffoni, Geneviève Blondin, Lucia Banci

**Affiliations:** ^1^ Magnetic Resonance Center CERM University of Florence Via L. Sacconi 6 50019 Sesto Fiorentino Florence Italy; ^2^ Consorzio Interuniversitario Risonanze Magnetiche di Metalloproteine (CIRMMP) Via L. Sacconi 6 50019 Sesto Fiorentino Florence Italy; ^3^ Laboratoire de Chimie et Biologie des Métaux Univ. Grenoble Alpes CNRS, CEA, IRIG, UMR 5249 17 rue des Martyrs 38000 Grenoble France; ^4^ Department of Chemistry University of Florence Via della Lastruccia 3 50019 Sesto Fiorentino Florence Italy

**Keywords:** anamorsin, in cellulo EPR spectroscopy, in cellulo Mössbauer spectroscopy, iron–sulfur clusters, metalloproteins

## Abstract

Human anamorsin is an iron–sulfur (Fe–S)‐cluster‐binding protein acting as an electron donor in the early steps of cytosolic iron–sulfur protein biogenesis. Human anamorsin belongs to the eukaryotic CIAPIN1 protein family and contains two highly conserved cysteine‐rich motifs, each binding an Fe–S cluster. In vitro works by various groups have provided rather controversial results for the type of Fe–S clusters bound to the CIAPIN1 proteins. In order to unravel the knot on this topic, we used an in cellulo approach combining Mössbauer and EPR spectroscopies to characterize the iron–sulfur‐cluster‐bound form of human anamorsin. We found that the protein binds two [2Fe–2S] clusters at both its cysteine‐rich motifs.

## Graphical abstract

A combined in cellulo Mössbauer‐ and EPR‐based approach is applied to address controversial aspects of Fe–S cluster‐binding properties of human anamorsin, a protein involved in the biogenesis of cytosolic [4Fe–4S] cluster‐binding proteins. We show that human anamorsin binds two [2Fe–2S] clusters in‐cell in two highly conserved cysteine‐rich motifs typical of the eukaryotic CIAPIN1 protein family, with different electron‐spin‐relaxation properties.

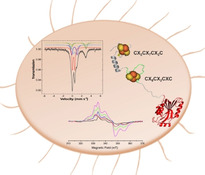

Iron–sulfur (Fe–S) clusters are ancient, polynuclear inorganic cofactors, containing iron ions (Fe^2+/3+^) and inorganic sulfide (S^2−^),^[1]^ present in all kingdoms of life. Proteins that bind Fe–S clusters are involved in many essential life processes, ranging from metabolic reactions to electron transport, DNA maintenance and gene expression regulation.[^2^, ^3^] Among the Fe–S binding proteins, the eukaryotic CIAPIN1 protein family is characterized by the presence of a N‐terminal S‐adenosyl methionine methyl transferase‐like domain connected via a flexible linker to a C‐terminal cytokine‐induced apoptosis inhibitor 1 (CIAPIN1) domain.[^4^, ^5^, ^6^] The hallmark of this protein family is the presence, in the CIAPIN1 domain, of two highly conserved cysteine‐rich motifs (a CX_8_CX_2_CXC motif (M1 motif, hereafter) followed by a CX_2_CX_7_CX_2_C motif (M2 motif, hereafter)), each able to bind an Fe–S cluster.[^6^, ^7^, ^8^, ^9^, ^10^] This protein in human,^[11]^ yeast,^[12]^ plant[^13^, ^14^, ^15^] and in the protist *Trypanosoma brucei* ^[16]^ has been proposed to act in the early stages of the cytoplasmic Fe–S protein biogenesis by working as an electron donor in an electron transfer chain required for the assembly of [4Fe–4S] clusters.

There is an ongoing debate in the literature on the type of Fe–S clusters bound to the CIAPIN1 proteins, which have been found indeed to bind [4Fe–4S] or [2Fe–2S] clusters, depending on the purification procedures used to isolate the holo species.[^6^, ^7^, ^8^, ^9^, ^10^, ^12^] In order to shed light on this matter, the Fe–S binding properties of human anamorsin were here investigated by *in cellulo* Mössbauer and *in cellulo* EPR spectroscopies. Specifically, wild‐type protein (WT‐anamorsin, hereafter) and a mutant containing only the M2 cysteine‐rich motif (M2‐anamorsin, hereafter), obtained by mutating the four cysteines of the M1 motif of anamorsin into alanines, were used to characterize the nature of the Fe–S clusters bound to anamorsin directly in cell.

Mössbauer spectra of *E. coli* cells expressing WT‐ and M2‐anamorsin in the presence of ^57^Fe and of the related control cells (see Supplementary Information for details) were recorded at ca. 6 K in a 60 mT external magnetic field applied parallel to the γ‐rays. The spectra recorded on the cells expressing M2‐ and WT‐anamorsin (Figure 1 A and 1 C, respectively) clearly showed two lines at ≈0.0 and ≈0.5 mm s^−1^. These lines were absent in the spectra recorded on the control cells samples (Figure 1 B and 1 D), that were similar to spectra previously reported for control cells.[^17^, ^18^, ^19^, ^20^, ^21^, ^22^] Spectra of control cells exhibited signatures from high‐spin ferrous species, that were clearly evidenced by the presence of the high velocity line at ≈3 mm s^−1^, whose absorption profile suggested the presence of two different components. The spectra also showed a signal between ≈0 and ≈1 mm s^−1^ corresponding to the combination of nanoparticles (NP), and of low‐spin ferrous heme and diamagnetic [4Fe–4S]^2+^ clusters, the latter two species presenting similar nuclear parameters, and usually denoted as the Central Doublet (CD). Accordingly, four doublets were considered to reproduce the spectra of control cells. A fifth doublet was introduced to fit the spectra of induced cells. This additional doublet corresponds to the difference spectrum obtained by subtracting the control cells spectrum from that of the induced cells (see Supporting Information, Figure S1). The simulations (see Supporting Information for details) and the experimental spectra are shown in Figure 1, where the different contributions of the Fe sites are displayed above each spectrum. The parameters used for the simulations are listed in Table 1.

**Figure 1:**
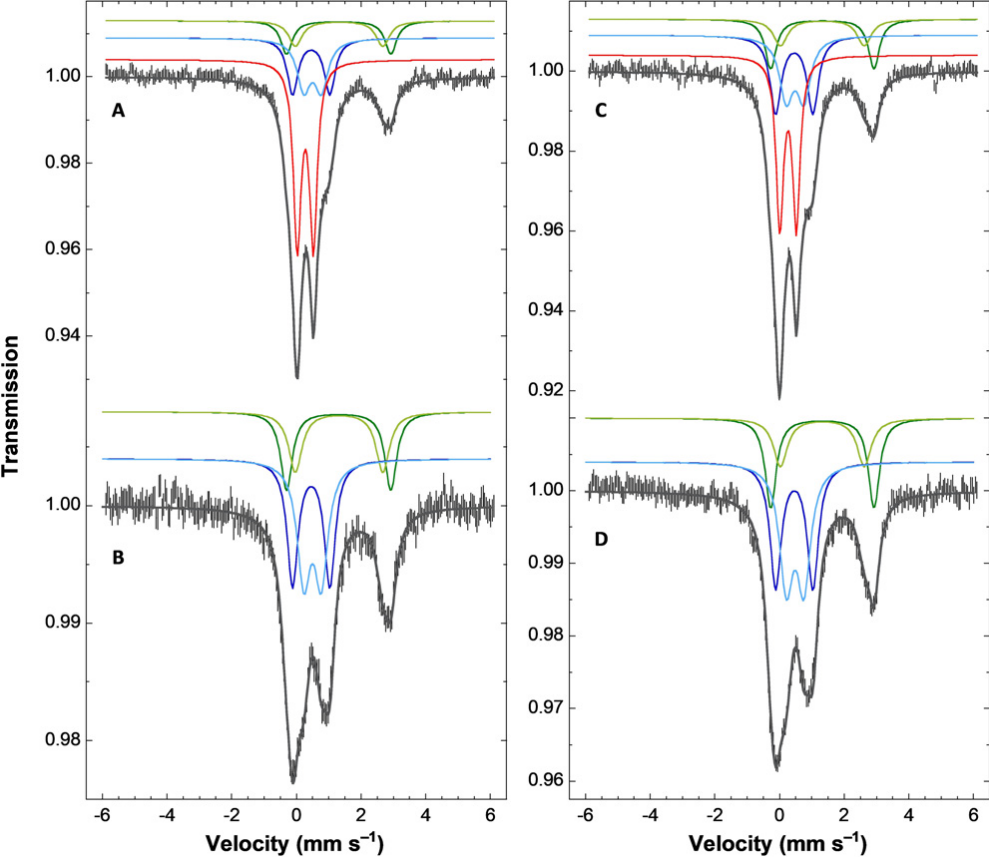
M2‐ and WT‐anamorsin expressed in E. coli cells bind [2Fe–2S]^2+^ clusters by Mössbauer spectroscopy. Experimental Mössbauer spectra (hatched bars) recorded at ca. 6 K on cells samples with (A and C) or without (B and D) induction of the expression of M2‐ (left panel) and WT‐anamorsin (right panel). A 60 mT external magnetic field was applied parallel to the γ‐rays. The grey solid lines are simulations of the spectra with parameters listed in Table 1. Contributions are displayed as solid lines above the spectra using the following color code: HS Fe^II^ (1) in green, HS Fe^II^ (2) in light green, CD in blue, NP in light blue, [2Fe–2S] in red.

**Table 1:** Parameters used for the simulated spectra shown as grey solid lines in Figure 1. Uncertainties are ±0.02, ±0.05, ±0.04, and ±4 for the isomer shift (δ), the quadrupole splitting (Δ*E*_Q_), the Lorentzian full‐width at half‐maximum (Γ), and the relative area, respectively.

Proteins	Fe sites^[a]^	δ [mm s^−1^]	Δ*E*_Q_ [mm s^−1^]	Γ [mm s^−1^]^[b]^	% in control cells^[c]^	% in induced cells
M2‐anamorsin	HS Fe^II^ (1)	1.30	3.22	0.44	20	11
	HS Fe^II^ (2)	1.31	2.71	0.50	18	10
	CD^d^	0.45	1.15	0.40	30	18
	NP	0.49	0.54	0.49	33	19
	[2Fe–2S]	0.27	0.50	0.28	0	41
WT‐anamorsin	HS Fe^II^ (1)	1.32	3.19	0.41	21	14
	HS Fe^II^ (2)	1.32	2.59	0.52	14	10
	CD^[d]^	0.45	1.15	0.42	30	22
	NP	0.47	0.54	0.50	34	21
	[2Fe–2S]	0.26	0.51	0.27	0	32

[a] The two HS Fe^II^ populations were assumed to be in the same ratio in the control and induced cells. [b] For each Fe site, a common linewidth was assumed in the control and induced cells spectra. [c] A zero contribution was fixed for the [2Fe–2S] clusters in control cells. [d] The Central Doublet (CD) reproduced the low‐spin ferrous hemes and the diamagnetic [4Fe–4S]^2+^ clusters. δ and Δ*E*_Q_ were fixed to their usual values.[^19^, ^23^]

The nuclear parameters of the additional Fe site detected upon induction were typical of oxidized [2Fe–2S]^2+^ clusters.[^17^, ^18^, ^24^] This Fe site accounted for 32 % and 41 % of the total iron content for WT‐ and M2‐anamorsin induced cells, respectively. The higher percentage of [2Fe–2S] cluster in M2‐anamorsin is in agreement with its higher expression level with respect to that of WT‐anamorsin, as detected by Western blot gel analysis (Figure S2). It is worth noticing that the iron distribution determined in the control cells was not modified upon the induction of protein expression. More specifically, the contribution of CD partly accounting for [4Fe–4S]^2+^ clusters, did not vary significantly upon expression of WT‐ and M2‐anamorsin (see Supporting Information Table S1). The observed 1–2 % variations found in CD contribution are more than ten times below the amount of [2Fe–2S]^2+^ clusters detected upon anamorsin expression. Consequently, the incorporation of a [4Fe–4S]^2+^ cluster in the M1 or M2 motif of WT‐anamorsin is essentially negligible, and it can be safely concluded that both WT‐ and M2‐anamorsin accommodate [2Fe–2S]^2+^ clusters.

In combination with *in cellulo* Mössbauer spectra, we acquired continuous wave (CW) *in cellulo* EPR spectra of *E. coli* cells expressing WT‐ or M2‐anamorsin and of the related control cells, all treated with 10 mM sodium dithionite under anaerobic conditions (Figure S3). The *in cellulo* EPR difference spectra (obtained as reported in the Supporting Information) of reduced *E. coli* cells expressing M2‐anamorsin exhibited a rhombic spectrum over a wide range of temperatures (Figure 2 A), indicating that the signal arises from a single paramagnetic species. The EPR signal experienced the highest intensity at 10 K and was significantly broadened above 70 K (Figure 2 A). The *in cellulo* EPR difference spectrum recorded at 10 K was readily simulated with a single set of principal g values of 2.016, 1.935, 1.890 (Figure S4 and Table S2). When the microwave power was increased from 0.5 mW to 5 mW at 10 K, or the temperature was further lowered to 5 K the signal was easily saturated (Figure 2 A).

**Figure 2:**
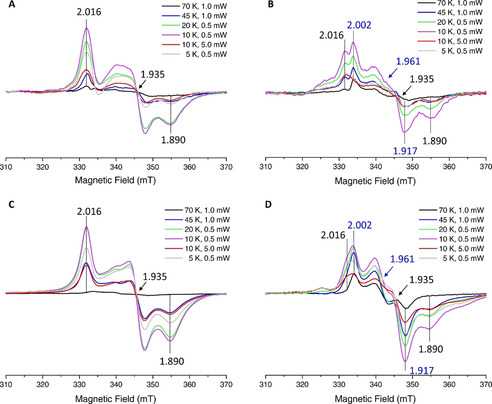
The M1‐ and M2‐motifs of anamorsin bind a [2Fe–2S] cluster by EPR spectroscopy. CW X‐band EPR spectra of reduced *E. coli* cells expressing A) M2‐anamorsin and B) WT‐anamorsin, at different temperatures and microwave powers, after subtraction of the spectra of the corresponding reduced control cells. EPR spectra of C) anaerobically purified M2‐anamorsin and D) anaerobically purified WT‐anamorsin after reduction with 10 mM sodium dithionite, at different temperatures and microwave powers. EPR spectra were recorded under the following conditions: microwave frequency, 9.36 GHz; modulation amplitude, 10 G; modulation frequency, 100 kHz; acquisition time constant, 163.84 ms; number of points 1024.

This behavior is consistent with the presence of a *S*=1/2 spin of a reduced [2Fe–2S]^+^ cluster that experiences a relatively slow relaxation rate at variance with the behavior of reduced [4Fe–4S]^+^ clusters. Indeed, the half‐integer electronic spin in a [4Fe–4S]^+^ cluster generally has much faster electron spin relaxation rates, and consequently their EPR signals are broadened beyond detection at temperatures around ≈25 K, while they are well detectable and hardly power saturated at lower temperatures.^[25]^ These features of the *in cellulo* EPR difference spectra of M2‐anamorsin thus indicate that the M2 motif coordinates a [2Fe–2S] cluster, in agreement with the *in cellulo* Mössbauer data. The electron spin relaxation properties of the reduced [2Fe–2S]^+^ cluster bound to M2‐anamorsin are, however, peculiar with respect to what is usually observed in ferredoxin‐type reduced [2Fe–2S]^+^ clusters, resembling in part those of fast relaxing reduced [4Fe–4S]^+^ clusters. Indeed, in ferredoxin‐type reduced [2Fe–2S]^+^ clusters the slow spin relaxation rates make the EPR signal hardly detectable below 10 K,^[25]^ contrarily to what observed for the reduced [2Fe–2S]^+^ cluster bound to M2‐anamorsin, whose EPR signal is still detectable at 5 K (Figure 2 A). This peculiar relaxation properties of the reduced [2Fe–2S]^+^ cluster bound to the M2 motif can be explained considering that the reduced cluster bound to the M2 motif revealed a valence localization‐to‐delocalization transition as a function of temperature, as previously described by us.^[7]^ It has been previously demonstrated that the electron delocalization within mixed‐valence Fe^II^Fe^III^ pairs favors the parallel alignment of the local spins of both the high‐spin Fe^II^ (*S* ${}_{\mathrm{Fe}{}^{\mathrm{II}}}$ =2) and high‐spin Fe^III^ (*S* ${}_{\mathrm{Fe}{}^{\mathrm{III}}}$ =5/2) ions, leading to a *S*=9/2 total spin ground state.^[26]^ The detection of the EPR signal of a *S*=1/2 spin suggests that the partial electron delocalization observed in the reduced [2Fe–2S]^+^ cluster bound to M2 motif is not strong enough to make the maximal total spin *S*=9/2 value as the ground state. However, it could allow the lowering in energy of the excited *S*>1/2 spin states, thus leading to a faster electron spin relaxation rate for the ground *S*=1/2 state compared with those typically observed in ferredoxin‐type [2Fe–2S] clusters, thus more closely mimicking an electron spin relaxation rate value typical of fast relaxing [4Fe–4S] clusters.

Reduced *E. coli* cells expressing WT‐anamorsin showed more complex *in cellulo* EPR difference spectra, arising from the presence of two rhombic EPR signals (Figure 2 B). The *in cellulo* EPR difference spectrum recorded at 10 K was readily simulated with two subspectra having principal g values of 2.002, 1.961, 1.917 and of 2.016, 1.935, 1.890 (Figure S4 and Table S2), that were previously assigned to the two [2Fe–2S]^+^ clusters bound to the M1 and M2 motifs of WT‐anamorsin, respectively.^[7]^ At 70 K, the *in cellulo* EPR difference spectrum was dominated by the signal arising from the *S*=1/2 spin of the [2Fe–2S]^+^ cluster bound to the M1 motif of WT‐anamorsin.^[7]^ By lowering the temperature to 10 K, the contribution of the signal originating from the M2‐bound [2Fe–2S]^+^ cluster increased, as showed by the increase in the intensity of the signal at *g*=2.016 and *g*=1.890 (Figure 2 B). As the microwave power was increased from 0.5 mW to 5 mW at 10 K, or the temperature was further lowered from 10 K to 5 K, the EPR signals of both clusters bound to WT‐anamorsin were easily saturated, although to a different extent, being the EPR signal of the M1‐bound [2Fe–2S]^+^ cluster more easily saturated than that of the M2‐bound [2Fe–2S]^+^ cluster, as showed by the larger decrease in the intensity of the signal at *g*=2.002 compared to that at *g*=2.016 in Figure 2 B. This behavior reproduces that observed for reduced *E. coli* cells expressing M2‐anamorsin (Figure 2 A) and is consistent with the binding of [2Fe–2S]^+^ clusters to both M1 and M2 anamorsin motifs, with very similar, low dispersed g values, but significantly different electron spin relaxation properties and thus different saturation characteristics, as already described above.

To investigate whether the Fe–S clusters of anamorsin are modified along the purification procedure, we recorded the EPR spectra of WT‐ and M2‐anamorsin anaerobically purified from *E. coli* cells and then treated with 10 mM sodium dithionite under anaerobic conditions. These EPR spectra exhibited rhombic signals with the same sets of g values, relaxation and power saturation characteristics of those observed for reduced *E. coli* cells expressing WT‐ and M2‐anamorsin, respectively (Figure 2 C and 2 D). These results clearly indicate that the clusters bound to human anamorsin in the cytoplasmic environment are conserved upon anaerobic purification.

It is remarkable to note that *in cellulo* EPR spectroscopy, at variance with *in cellulo* Mössbauer spectroscopy, is able to spectroscopically discern the presence of two *S*=1/2 spins arising from [2Fe–2S]^+^ clusters bound to the M1 and M2 motifs of WT‐anamorsin. Thus, *in cellulo* EPR data clearly demonstrate that M1‐ and M2‐sites of WT‐anamorsin are both occupied by [2Fe–2S] clusters in *E. coli* cells. The differences in the electronic properties allowing the distinction between the two reduced clusters are suppressed in the oxidized state, which features a diamagnetic ground state. Furthermore, the isomer shift for the ferric sites in an oxidized [2Fe–2S]^2+^ cluster is only moderately sensitive to the ligand environment. To the best of our knowledge, only the presence of one or two histidines in place of cysteines in the coordination sphere leads to a significant increase of *δ* (ref. [27] and references therein).

Here, we have shown by *in cellulo* Mössbauer and *in cellulo* EPR spectroscopies that, at variance with what reported for yeast Dre2 that was described to bind a [2Fe–2S] cluster at the M1 motif and a [4Fe–4S] cluster at the M2‐motif, human anamorsin coordinates two [2Fe–2S] clusters, one in each M1 and M2 motif. In addition, EPR spectra acquired on both reduced *E. coli* cells expressing WT‐ and M2‐anamorsin and on anaerobically purified, reduced WT‐ and M2‐anamorsin showed that the [2Fe–2S] cluster bound to the M2 motif of anamorsin displays enhanced electron spin relaxation rates, likely originating from local protein conformational heterogeneity.^[7]^ Our study, showing that *in cellulo* anamorsin binds two [2Fe–2S] clusters at both M1 and M2 motifs, is consistent with the hypothesis that this holo form of anamorsin is the physiologically relevant species. Our data also showed that the reducing environment of the bacterial cytoplasm, and presumably also that of the human cytoplasm, is not sufficient to reduce the [2Fe–2S]^2+^ clusters of anamorsin, but that a reductase is required to activate anamorsin function as cellular reductant to assemble [4Fe–4S] clusters in the early steps of cytosolic Fe–S protein biogenesis. The NADPH‐dependent diflavin oxidoreductase 1 (Ndor1), which is responsible, in eukaryotic cells, for the reduction of the [2Fe–2S] cluster bound to the M1‐site of anamorsin[^7^, ^11^, ^12^], is, however, not present in bacterial organisms, and thus this explains why the M1‐bound [2Fe–2S] cluster of anamorsin is exclusively present in an oxidized state, when expressed in *E. coli* cells.

## Conflict of interest

The authors declare no conflict of interest.

## Supporting information

As a service to our authors and readers, this journal provides supporting information supplied by the authors. Such materials are peer reviewed and may be re‐organized for online delivery, but are not copy‐edited or typeset. Technical support issues arising from supporting information (other than missing files) should be addressed to the authors.

Supplementary
