# Supplementary material for: In Cellulo Mössbauer and EPR Studies Bring New Evidence to the Long‐Standing Debate on Iron–Sulfur Cluster Binding in Human Anamorsin
Source: Angew Chem Int Ed Engl. 2021 May 24;60(27):14841–5. doi: 10.1002/anie.202102910 (PMC8251831; doi:10.1002/anie.202102910)
Supplement: Supplementary file 1 — Supplementary [file ANIE-60-14841-s001.pdf]

Author Contributions

S.M. Investigation: Equal; Validation: Equal
F.C. Investigation: Equal; Validation: Equal; Visualization: Lead; Writing—original draft: Equal
M.C. Formal analysis: Equal; Investigation: Equal; Validation: Equal
S.C.B. Conceptualization: Equal; Supervision: Equal; Writing—review & editing: Equal
G.B. Funding acquisition: Equal; Investigation: Equal; Supervision: Lead; Visualization: Equal; Writing—original draft: Equal
L.B. Conceptualization: Equal; Funding acquisition: Equal; Project administration: Lead; Supervision: Equal; Writing—review & editing: Equal.
